# Increased incidence of weight-loss-associated humane endpoints in rats administered buprenorphine slow-release LAB formulation following traumatic brain injury: a retrospective study

**DOI:** 10.3389/fneur.2024.1467419

**Published:** 2024-09-23

**Authors:** Radina L. Lilova, Martina Hernandez, Corrina Kelliher, Audrey Lafrenaye

**Affiliations:** ^1^Department of Anatomy and Neurobiology, Virginia Commonwealth University School of Medicine, Richmond, VA, United States; ^2^Department of Neurology, Duke University School of Medicine, Durham, NC, United States

**Keywords:** buprenorphine slow-release LAB, buprenorphine slow-release HCl, traumatic brain injury, rats, weight loss, humane endpoints

## Abstract

Traumatic brain injury (TBI) remains a significant global public health epidemic with adverse health and cost implications. Due to its complex, heterogeneous nature and wide-ranging impacts, definitive TBI treatments remain elusive. As such, continued laboratory research using animal models is warranted. In accordance with guidelines set forth for the humane treatment of research animals, TBI animal models are often administered analgesics for pain management. The choice of drug, timing, dose, and formulation of analgesic can vary depending on the study’s unique needs and can potentially and unintentionally influence experimental results. In TBI studies utilizing rats as animal models, buprenorphine is a common analgesic administered. In addition to pain management in such studies, investigators must also monitor the research animals post-operatively and make the decision for humane euthanasia before intended experimental survival timepoint if the animals are assessed to be excessively suffering. This study investigated the differences in adult, male Sprague Dawley rats used for various TBI studies that reached weight-loss-induced humane endpoints following a single administration of buprenorphine slow-release LAB (bup-SR-LAB) or buprenorphine slow-release HCl (bup-SR-HCl). Our findings indicate that TBI-induced rats receiving bup-SR-LAB in conjunction with a secondary surgical insult such as artificial intracranial pressure elevation and/or osmotic pump implantation reach a weight-loss-induced humane euthanasia endpoint more often compared to sham-injured rats. When stratifying into the same groups, we did not find this pattern to hold true for rats administered bup-SR-HCl. Overall, this study contributes to the limited body of literature addressing different analgesic formulations’ effects on laboratory animals.

## Introduction

Traumatic brain injury (TBI) is a significant global public health burden with an estimated yearly incidence between 27 to 69 million people and with no definitive cure (1–3). It is a complex and highly heterogenous condition that can result in a wide range of cognitive (4–6), emotional (7–9), sensory (10), and physical (11–13) impairments that can be both acute and chronic (14–16). The complexity of TBI pathophysiology has thus far prevented effective treatments from being developed, underscoring a need for continued research. To better understand the underlying mechanisms of TBI and develop effective therapeutics, various pre-clinical animal models have been established for research use. Such models are beneficial for characterizing and manipulating molecular mechanisms and chemical cascades that occur post-injury.

When conducting TBI survival studies, particularly those involving longer-term experimental timepoints, an additional variable that must be considered is the appropriate pain management of the animals (17–20). Depending on the species and interventions performed, different drugs, formulations, routes of administration, and dosages are utilized (18, 21). In TBI procedures utilizing rats, buprenorphine is an effective and commonly used analgesic (22, 23). Buprenorphine is a high-affinity partial agonist at the mu-opioid receptor as well as an antagonist at the kappa-opioid receptor, providing analgesia with lower risks of respiratory depression and sedation compared to full opioid agonists (24). Previous studies have shown mixed results regarding the impact of buprenorphine on various physiological and behavioral parameters in animal models, with some indications for potential effects on outcomes in TBI research (25–29). Our group has previously reported that a single dose of slow-release buprenorphine administered once following induction of a central fluid percussion injury (cFPI) to the brain in rats precipitates glial cell morphological changes (30, 31). However, as a whole the different analgesic impacts on research animals undergoing a variety of procedures, both TBI and elsewise, remain underreported on.

The current study is a secondary retrospective analysis of rats from various TBI studies in our lab receiving two different formulations and veterinarian-recommended dosages of buprenorphine, buprenorphine slow-release LAB (bup-SR-LAB) and buprenorphine slow-release HCl (bup-SR-HCl). During the execution of these previous studies, it was observed that a higher-than-expected number of animals were reaching humane endpoints due to weight loss after procedures. In the current study, we investigated rats administered buprenorphine following sham injury or cFPI-induced TBI that reached a humane endpoint due to weight loss, thereby requiring euthanasia before intended experimental endpoint.

## Methods

### Animals

This study is a retrospective analysis of animals generated for other TBI studies. All animal studies were conducted in accordance with ARRIVE guidelines (32) and approved by the Institutional Animal Care and Use Committee at Virginia Commonwealth University (approval #AM10251), which adhere to regulations including, but not limited to, the Virginia Commonwealth University institutional ethical guidelines concerning the care and use of laboratory animals, and those set forth in the *Guide for the Care and Use of Laboratory Animals: 8th Edition* (33). Adult (12-16-week-old), male Sprague Dawley rats were included in this study. Animals were housed in individual cages on a 12-h light–dark cycle with free access to food and water as well as full veterinary oversight.

### Animal inclusion

**Figure 1:**
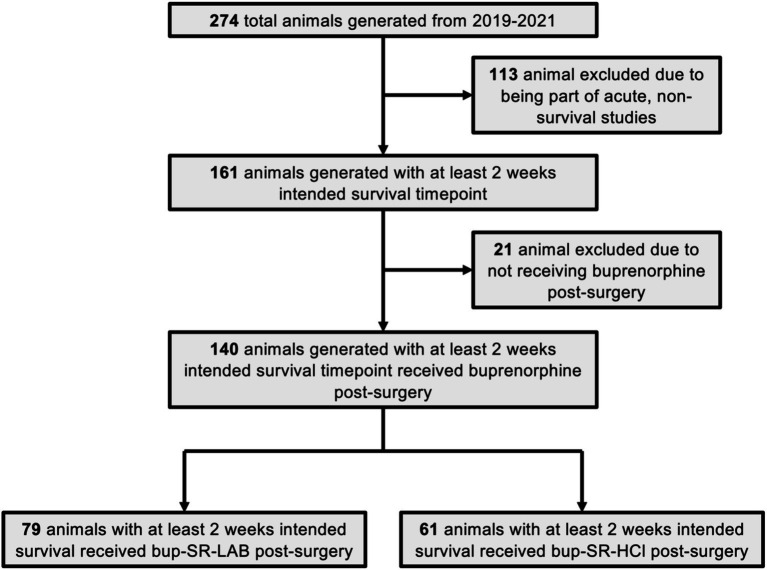
Flow chart of study inclusion and exclusion criteria. This flow chart depicts the total number of Sprague Dawley rats generated during the period of analysis from January 2019 through December of 2021. Rats generated for non-survival studies and rats not administered buprenorphine after surgery were excluded from this analysis. The final number of animals eligible to be included in the study were divided into the two main cohorts of interest: those receiving the bup-SR-LAB formulation and those receiving the bup-SR-HCl formulation administered subcutaneously at 15 min post-surgery.

All animals used in this analysis were originally generated for and were part of other studies, some of which have been incorporated in previously published works (31, 34, 35). During the time from January 1, 2019, through December 31, 2021, a total of 274 animals were generated by the laboratory and 140 met initial inclusion criteria for this analysis (Figure 1). Included animals were all administered either bup-SR-LAB (1 mg/mL; ZooPharm, Laramie, WY, United States) from January 1, 2019, through December 31, 2020, or bup-SR-HCl (1 mg/mL; ZooPharm, Laramie, WY, United States) from January 1, 2021, through December 31, 2021. All bup-SR doses were administered subcutaneously (SC) 15 min post-injury. No other notable changes were made to surgical preparations, procedures, or animal care during this time.

The five experimental design groups used for this retrospective investigation encompassed all general conditions of the animals the laboratory generated. The five groups were as follows: group (1) sham-injured controls (Sham; bup-SR-LAB *n* = 13, bup-SR-HCl *n* = 3); group (2) sham-injured animals with osmotic pump implantation (Sham + Implant; bup-SR-LAB *n* = 10, bup-SR-HCl *n* = 12); group (3) animals sustaining only cFPI (TBI; bup-SR-LAB n = 17, bup-SR-HCl *n* = 4); group (4) animals sustaining cFPI followed by osmotic pump implantation and secondary ICP elevation (TBI + ICP + Implant; bup-SR-LAB *n* = 16, bup-SR-HCl *n* = 14); group (5) animals sustaining cFPI followed by either osmotic pump implantation or secondary ICP elevation (TBI + ICP or Implant; bup-SR-LAB *n* = 23, bup-SR-HCl *n* = 28). For all studies included in this retrospective analysis, a random number generator determined animal group prior to surgery.

### Surgical preparation, injury induction, and drug administration

Methodology for surgical preparation and injury induction of animals used for this retrospective study have been published previously (31, 34, 35). Briefly, anesthesia was induced with 4% isoflurane in 30% O_2_/70% room air. Animals were ventilated with 1.5–2.5% isoflurane in 30% O_2_/70% room air throughout surgery duration, injury, and post-injury ICP elevation and/or osmotic pump implantation. Body temperature was maintained at 37°C with a rectal thermometer connected to a feedback-controlled heating pad (Harvard Apparatus, Holliston, MA, United States). Animals were placed in a stereotaxic frame (David Kopf Instruments, Tujunga, CA, United States) and a 4.8 mm diameter circular craniectomy was made along the sagittal suture midway between bregma and lambda, leaving the dura intact. Following the central craniectomy, a 2 mm diameter burr hole was drilled over the left lateral ventricle positioned at 1.3 mm lateral, 0.8 mm posterior, and 2.5–3 mm ventral to bregma. A 25G canula connected was to a pressure transducer and an 11 Elite microinfusion pump (Harvard Apparatus) in a closed, fluid-filled system was advanced into the lateral ventricle to measure intracranial pressure (ICP). Bone wax was used to seal the burr hole used for ICP measurements before preparation for central fluid percussion injury (cFPI). Procedures used to induce cFPI were consistent with those described previously (31, 34, 36, 37). Briefly, a Luer-Lok^™^ syringe (BD, Franklin Lakes, NJ, United States) hub was affixed to the craniectomy site with methyl methacrylate (Hygenic, Akron, OH, United States) applied around the hub and was allowed to harden. Animals were removed from the stereotaxic frame and placed on a raised platform for connection to the fluid percussion device, maintaining an unbroken fluid-filled system from the intact dura through the cylinder via an adaptor. To induce a mild-to-moderate cFPI, a pendulum was released onto the end of the fluid-filled cylinder of the percussion device, producing a pressure pulse that reached the animal at 2.05 ± 0.10 atmospheres for a duration of approximately 22.5 ms. This pressure pulse was transduced through the intact dura to the CSF and spread diffusely throughout the brain. The magnitude of the pressure pulse was measured via transducer affixed to the injury device and output on an oscilloscope display (Tektronix, Beaverton, OR, United States). For sham-injured animals, the same procedures were performed except the final pendulum release. Immediately following injury, animals were reconnected to the ventilator and the hub, dental acrylic, and bone wax were removed *en bloc* and gel foam was placed overtop the craniectomy site to stop bleeding. The animals were replaced in the stereotaxic frame and the ICP probe was reinserted into the lateral ventricle for post-injury ICP monitoring or manual ICP elevation. For animals undergoing manual ICP elevation to 15, 20, or 25 mmHg, infusion of sterile normal saline at a rate of 1.3–13 μL/min was performed. Once a target ICP was achieved, it was maintained at a steady state for 1 h. Fifteen min following the sham injury or cFPI, buprenorphine (1 mg/kg of a 1 mg/mL bup-SR-LAB solution or 0.9 mg/kg of a 1 mg/mL solution of bup-SR-HCl from ZooPharm, Laramie, WY, United States) was administered subcutaneously, with dosages following veterinarian recommendations. One hour following the sham injury or cFPI, animals in groups 2, 4, and 5 underwent implantation of a mini osmotic pump into the subcutaneous neck facia. A canula was run from the osmotic pump to a brain infusion adaptor implanted into the ICP burr hole for infusion into the left lateral ventricle.

Following recovery, rats were returned to individual housing in clean home cages. Comprehensive postoperative monitoring was done to obtain a more complete picture of animal recovery in which a three-category moribundity score was used. Specifically, animals were assessed for (1) general appearance (dehydration, decreased body weight < 20%, abnormal posture, swelling, prolapses), (2) skin/fur appearance (discoloration, urine stain, pallor, redness, cyanosis, wounds, sores, abscesses, ulcers, ruffled fur), and (3) locomotion (hyperactivity, lethargy, coma, ataxia, tremors). Each category was scored from 0 (best score) to 2 (worst score). Animals were assessed using this scale and weighed daily for at least 3 days post-surgery and weekly thereafter. Animals raising any humane concerns were monitored more frequently, being weighed and assessed daily until humane concerns resolved or until a humane endpoint was reached.

### Statistics

All statistical analyses were conducted using R (*R4.3.3*) (38). Pairwise Fisher exact tests were used for comparisons between groups. Statistical significance threshold was set at *p* = 0.05.

## Results

### Inclusion/exclusion

Of the 274 animals generated by the laboratory during the defined analysis period from January 1, 2019, through December 31, 2021, a total of 140 animals met initial inclusion criteria for this retrospective analysis (Figure 1). One hundred and thirteen animals were excluded from this assessment due to being part of non-survival studies or acute studies in which the animals’ experimental endpoint was intended for less than 2 weeks post-cFPI. A total of 21 animals incorporated into Ryu et al. Two thousand and twenty two were not administered any form of bup-SR and were therefore excluded from this study (31). Ultimately, 79 animals administered a vet-recommended dose of SC bup-SR-LAB (1 mg/kg), and 61 animals administered a vet-recommended dose of SC bup-SR-HCl (0.9 mg/kg) 15 min post-cFPI had experimental endpoints of at least 2 weeks post-injury and met all appropriate inclusion criteria to be stratified for further analysis (Figure 1).

### Weight loss assessment

As stated previously, all animals were weighed on day of surgery prior to undergoing planned procedures. Mean pre-surgery weight (i.e., day 0) was 428 g with a standard deviation of 54.4 g (Supplementary Figure S1). Assessments for humane euthanasia were made during daily welfare checks using aforementioned criteria. Specifically, 77% (17/22) of the humane endpoint bup-SR-LAB animals had weight loss noted as a primary driver for humane euthanasia. The reasons for humane endpoint for the remaining bup-SR-LAB animals were suture integrity issues, 14% (3/22), and procedural complications, 9% (2/22). All animals receiving bup-SR-HCl that were euthanized prematurely had weight loss concerns primarily contributing to humane endpoints.

**Figure 2:**
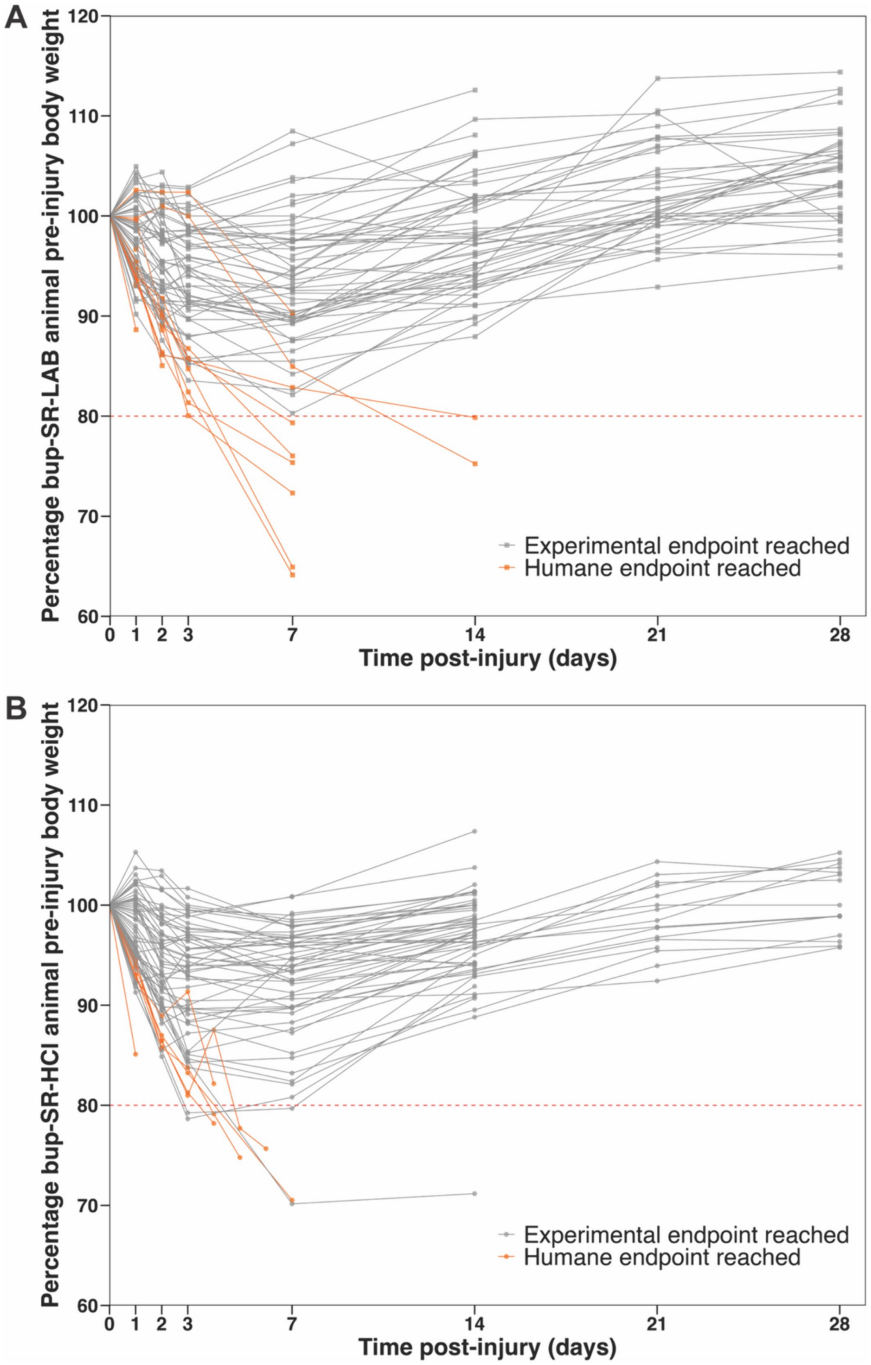
Reductions in the percentage of pre-injury body weight following TBI reached humane endpoints for a subset of animals administered bup-SR-LAB or bup-SR-HCl. Line grants depicting the percentage of pre-surgery body weight for all rats included in this analysis administered either **(A)** bup-SR-LAB or **(B)** bup-SR-HCl. Each line represents an individual rat. Points (squares bup-SR-LAB and circles bup-SR-HCl) indicate the percentage of pre-surgery body weight at 0, 1, 2, 3, 7, 14, 21, and 28 days post-surgery. Lines terminate at the animal’s final weight taken prior to euthanasia. Dashed red line indicates pre-determined humane weight-loss threshold endpoint, set at ≥80% body weight loss after surgery. Gray lines represent rats that survived as intended, reaching experimental endpoints. Orange lines represent rats that met criteria for humane endpoints.

Of the 74 total rats administered bup-SR-LAB, 77% (57/74) survived the entirety of the intended study period without weight-associated humane concerns (Figure 2A). Eight bup-SR-LAB rats (11%) dropped below 80% of their pre-injury body weight, which was the humane weight-loss threshold. An additional nine bup-SR-LAB rats (12%) did not reach humane weight-loss threshold of 80%, but did have weight loss trajectories that, when coupled with other observed humane indications, resulted in humane endpoints. Of the 61 total rats administered bup-SR-HCl, 89% (54/61) survived the entirety of the intended study period without humane concerns (Figure 2B). Four bup-SR-HCl rats (7%) dropped below 80% of their pre-injury weight. An additional three bup-SR-HCl rats (5%) did not reach the weight-loss humane threshold but had weight loss trajectories that, coupled with other humane concerns, resulted in humane endpoints (Figure 2B).

### Terminal endpoint

**Figure 3:**
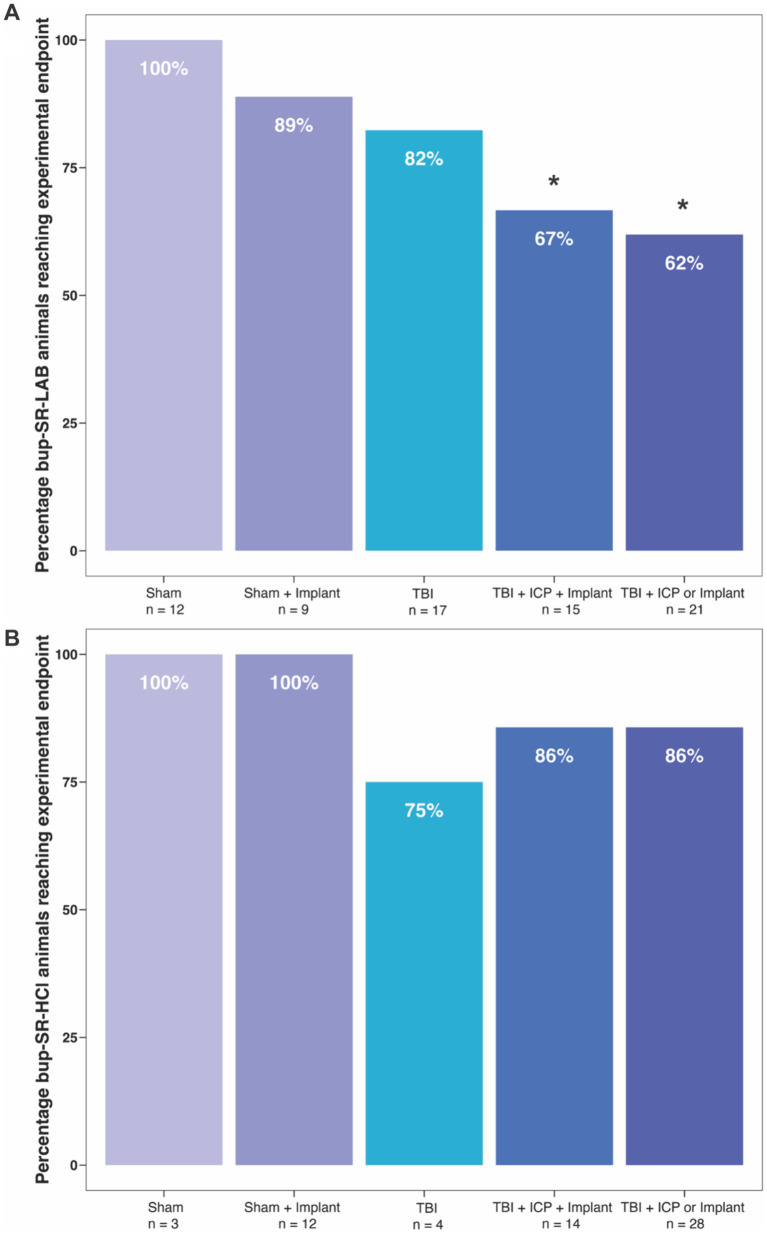
Lower percentage of rats administered bup-SR-LAB following TBI paired with an osmotic pump implantation and/or ICP elevation survived to experimental endpoints. Bar graphs representing the percentage of animals that survived to their experimental endpoint administered **(A)** bup-SR-LAB or **(B)** bup-SR-HCl. *p < 0.05 compared to sham.

Based on study designs conducted from 2019 to 2021, rats receiving either bup-SR-LAB or bup-SR-HCl were stratified into the five groups described in more detail previously: (1) Sham, (2) Sham + Implant, (3) TBI, (4) TBI + ICP + Implant, and (5) TBI + ICP or Implant. Upon stratifying bup-SR-LAB rats into these groups, it was found that the majority of humanely euthanized rats with significant weight loss concerns were given a cFPI paired with another insult, either osmotic pump implantation, secondary ICP elevation, or both (Figure 3A; Supplementary Figure S2A). Specifically, while 100% of Sham animals, 89% of Sham + Implant animals, and 82% of TBI animals survived until experimental endpoint, significantly fewer bup-SR-LAB rats within the TBI + ICP + Implant group (67%, *p* = 0.047 vs. Sham) or the TBI + ICP or Implant group (62%, *p* = 0.030 vs. Sham) survived to experimental endpoint (Figure 3A; Supplementary Figure S2A). This decrease in survival was not observed in animals administered bup-SR-HCl, as all groups maintained experimental endpoint survival rates at or above 75% (Figure 3B; Supplementary Figure S2B).

## Discussion

In TBI research as well as in other fields, the opioid buprenorphine is a common analgesic choice given to model animals (21). Extended-release or slow-release formulations are preferred in many cases due to longer acting time courses, which are reported to last up to 72 h (22, 39, 40), thus allowing for the administration of fewer doses while still adequately addressing the animals’ postoperative pain. While many researchers use bup-SR in their studies, few published reports exist regarding the impact different bup-SR formulations May have on investigations of TBI. Due to alternations in our study design, in 2019 we began to administer a single veterinarian-recommended dose of bup-SR-LAB for pain relief. Specifically, the studies that the rats were involved in required the implantation of osmotic pumps into the lateral ventricles and artificial increases in ICP following sham injury or cFPI-induced TBI. From 2019 through the end of 2020, we observed that a higher-than-expected number of animals were reaching humane endpoints due to weight loss. After ensuring that protocols and procedures were appropriately being carried out by all surgeons, input from the overseeing veterinarian led the team to switch the post-operative analgesic from 1 mg/kg bup-SR-LAB to 0.9 mg/kg bup-SR-HCl in January of 2021. While some animals did still reach humane endpoints due to weight loss concerns, a notable decrease in amount was observed, prompting this study. Therefore, we conducted a retrospective analysis investigating the proportion of rats given bup-SR-LAB or bup-SR-HCl following varying TBI interventions that reached a humane endpoint due to weight loss.

Because the different formulations of bup-SR were given in sequence and not in parallel, we did not directly compare the groups of animals between given bup-SR formulations. Therefore, we binned animals into five experimental design groups to assess if increase in weight-related humane endpoints was related to a combination of the types of procedures the rats underwent and the bup-SR formulation given. Groupings were made as specific as possible given the experiments performed. We found that rats administered bup-SR-LAB post-TBI paired with ICP elevation and/or osmotic pump implantation were particularly vulnerable to weight-related humane endpoints (Figures 2A, 3A). This increased vulnerability was not observed in rats administered bup-SR-HCl (Figures 2B, 3B). The nature of secondary, retrospective analyses such as this imparts certain limitations. Since the animal studies were designed and powered for a variety of other experiments and not a head-to-head assessment between the two bup-SR formulations’ effects on animal weight, the number of animals within the five groups were not equal. As such, the analysis May not be adequately powered to robustly reveal all differences between groups.

Since the surgeries were all performed by the same group of surgeons in the same laboratory using the same equipment, methods, and general protocols, the likelihood of such factors significantly influencing data are minimal. All surgeons were trained to levels of proficiency and the lab has extensive experience using the cFPI model. An additional factor to consider with potential to contribute to survival differences is the seasonal effect on laboratory animals, with effects previously shown (41–45). However, given the inclusion criteria dates spanned entire calendar years and animals were operated on consistently throughout the year, such potential seasonal biases are minimized. An additional consideration was the decrease in dose from 1 mg/kg bup-SR-LAB to 0.9 mg/kg bup-SR-HCl. Based on pre-surgical weights of the heaviest (694 g) and lightest (339 g) rats, the maximum administration difference between bup-SR-HCl and bup-SR-LAB was between 0.03 and 0.07 mg. This 10% difference in the dose was not assessed to be a significant enough contributor to result in the overall differences in humane endpoints observed (46–49). Other factors that May influence outcomes differences observed are surgical complications and/or infections. While a few rats incurred surgical complications requiring humane euthanasia, these complications were distinct from weight loss concerns and these animals were removed from analysis. Due to maintained consistency across the majority of study components, data presented here are valid and appropriate for use in the types of analyses reported.

While experiments were appropriately powered for the original, unfortunately we do not have the appropriate samples or data collected to more vigorously investigate the potential underlying reasons for the increased number of rats reaching weight-loss-induced humane endpoints with the bup-SR-LAB formulation versus the bup-SR-HCl formulation. While previous studies in our laboratory indicated a positive impact on weight with administration of bup-SR-LAB when compared with saline (30), these studies did not include groups with TBI and a secondary surgical insult, which were the ones found to be at-risk in this analysis. As such, those results are not contrary but rather additive to the ones reported here. Additionally, we do not know which compounds that exist in the bup-SR-LAB are not present in the bup-SR-HCl, and therefore which compounds May be contributing to the increased humane endpoints we observed. Overall, studies such as this help further inform the ever-growing field of TBI researchers on how analgesics could potentially (and unintentionally) influence data.

## Conclusion

Anesthesia and analgesia are administered to laboratory animals to control their perioperative pain for humane animal welfare reasons as well as for reducing the likelihood of introducing confounding factors brought on by uncontrolled pain and distress that May influence the studies (21). In the context of multifaceted traumatic brain injury research using animal models, the type of analgesic administered is also a consideration due to potential effects on behavior, central nervous system cell types, and post-surgical recovery (25–31). As such, using the most appropriate analgesics for the species and study type is a prime concern when designing and executing animal research studies. While reducing animals’ pain is critical, the formulation of the chosen analgesic is also an important decision point. Historically, particularly with rodent studies, analgesic practices have not been well-documented in literature, so with recent advances in transparency, more potential insights May be gleaned, and methods could be further refined (50, 51). The current study underscores the importance of appropriate and well-informed analgesic selection to limit unintended impacts on laboratory animals and, consequently, on the experiments as a whole.

## Data Availability

The raw data supporting the conclusions of this article will be made available by the authors, without undue reservation.
