# Supplementary material for: Increased incidence of weight-loss-associated humane endpoints in rats administered buprenorphine slow-release LAB formulation following traumatic brain injury: a retrospective study
Source: Front Neurol. 2024 Sep 23;15:1467419. doi: 10.3389/fneur.2024.1467419 (PMC11456484; doi:10.3389/fneur.2024.1467419)
Supplement: Supplementary file 1 [file Data_Sheet_1.docx]

Increased incidence of weight-loss-associated humane endpoints in rats administered buprenorphine slow-release LAB formulation following traumatic brain injury: a retrospective study

Radina L. Lilova^1^, Martina Hernandez^1^, Corrina Kelliher^1^, Audrey Lafrenaye^1*^

^1^Virginia Commonwealth University School of Medicine, Department of Anatomy and Neurobiology, Richmond, Virginia, United States of America

*** Correspondence:**Audrey Lafrenaye, email: Audrey.Lafrenaye@vcuhealth.org

Supplementary Material

## Supplementary Figures

##
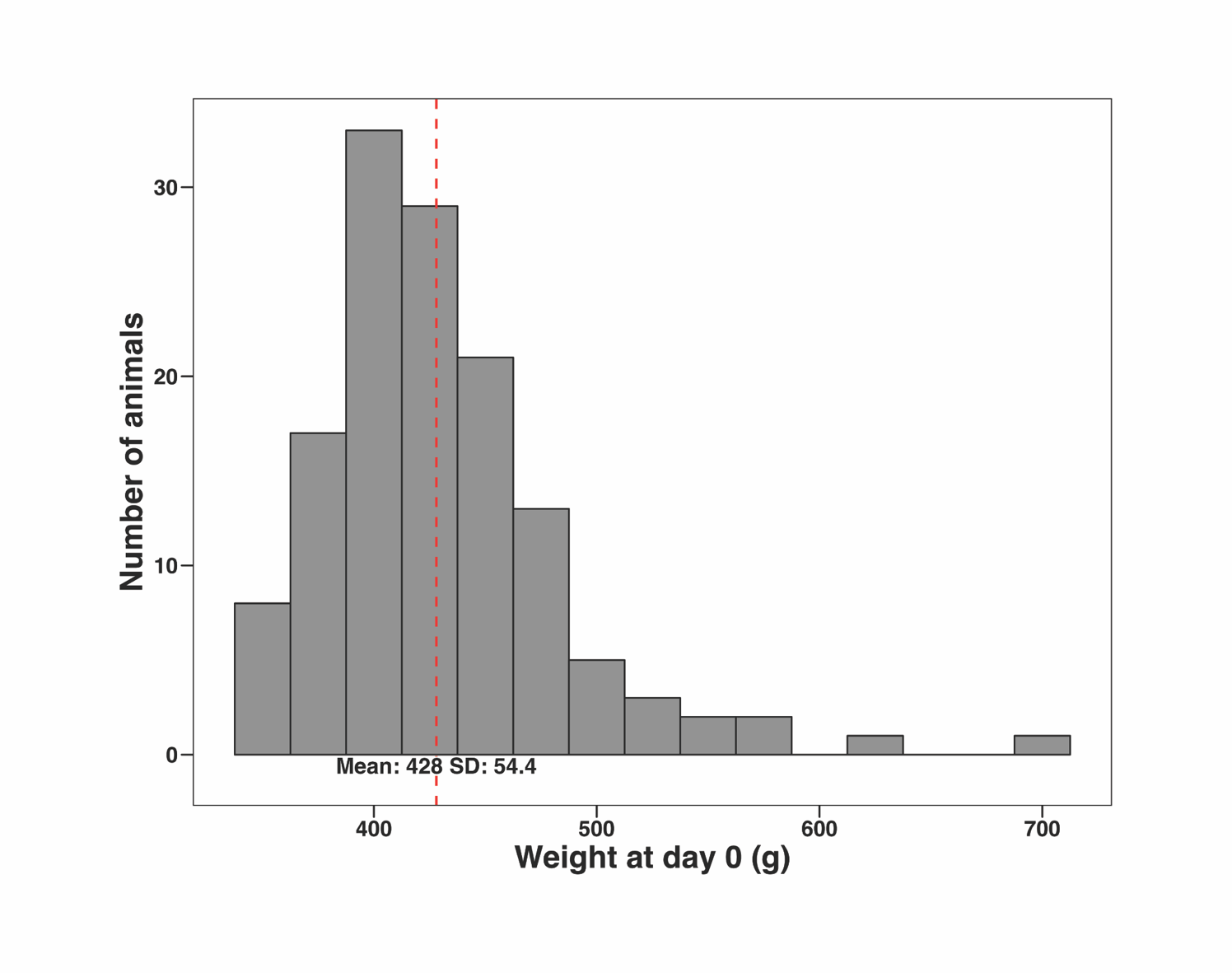


**Supplemental Figure 1. Pre-surgical weights of male rats included in this study.** Histogram of the starting weights of all n = 135 animals included in the final analyses. The red dotted line indicates the mean weight of animals used in this study.


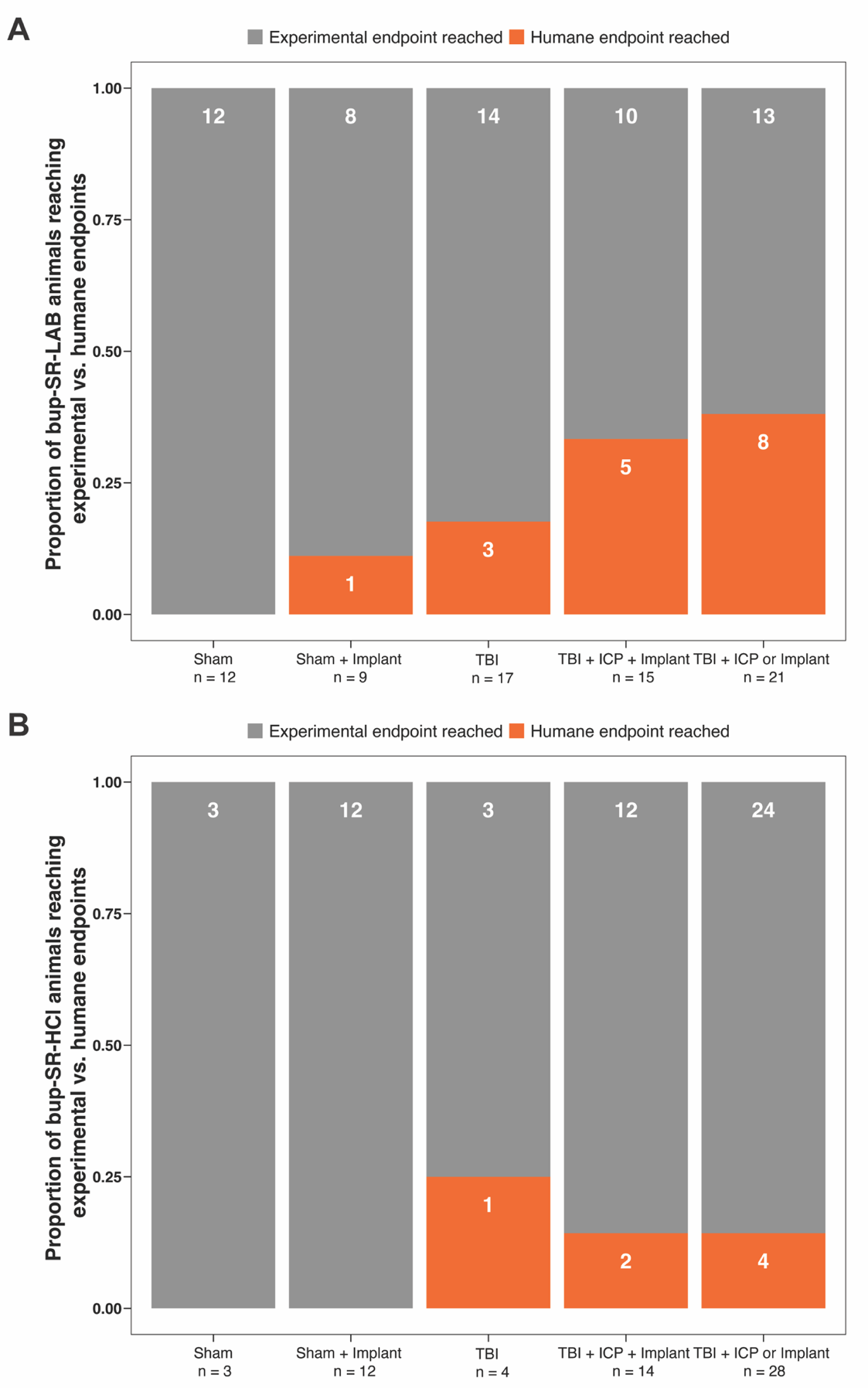


**Supplemental Figure 2. Proportion of rats reaching human endpoints by group.** Stacked bar graphs representing the ratio of animals administered (**A**) bup-SR-LAB or (**B**) bup-SR-HCl stratified by surgical group that maintained experimental endpoints (gray) and animals that reached weight-loss-associated humane endpoints (orange). Numbers in each stack represent the number of animals.
